# Dietary inflammatory potential is associated with sarcopenia in patients with hypertension: national health and nutrition examination study

**DOI:** 10.3389/fnut.2023.1176607

**Published:** 2023-05-12

**Authors:** Jiabin Tu, Shanshan Shi, Yuchen Liu, Jiaming Xiu, Yanbin Zhang, Bo Wu, Ying Liao, Kaihong Chen, Ganyang Li, Llling Chen

**Affiliations:** Longyan First Affiliated Hospital of Fujian Medical University, Longyan, China

**Keywords:** hypertension, sarcopenia, dietary inflammatory potential, NHANES, inflammatory

## Abstract

**Background:**

Study has shown that sarcopenia increases the risk of poor outcomes in patients with hypertension. Inflammation is one of the important reasons for the occurrence and development of sarcopenia. Regulating systemic inflammation may be a potential intervention for sarcopenia in hypertensive patients. Diet is one of the important measures to improve systemic inflammation. The dietary inflammatory index (DII) is a tool designed to assess the inflammatory potential of the diet, the association between DII and sarcopenia in hypertensive patients is unclear.

**Objective:**

To explore the relationship between the DII and sarcopenia in patients with hypertension.

**Method:**

Data from the National Health and Nutrition Examination Survey (NHANES) 1999–2006 and 2011–2018. A total of 7,829 participants were evaluated. Participants were divided into four groups based on the quartile of the DII: Q1 group (*n* = 1,958), Q2 group (*n* = 1,956), Q3 group (*n* = 1,958) and Q4 group (*n* = 1,957). The relationship between the DII and sarcopenia was assessed by logistic regression analysis based on the NHANES recommended weights.

**Result:**

The DII was significantly associated with sarcopenia in patients with hypertension. After full adjustment, patients with higher DII (OR: 1.22, 95% CI: 1.13–1.32, *p* < 0.001) have a higher risk of sarcopenia. Compared with Q1 group, the group with higher DII levels had a higher risk of sarcopenia (Q2: OR: 1.23, 95%CI: 0.89–1.72, *p* = 0.209; Q3: OR: 1.68, 95%CI: 1.20–2.35, *p* = 0.003; Q4: OR: 2.43, 95%CI: 1.74–3.39, *p* < 0.001).

**Conclusion:**

High DII is associated with an increased risk of sarcopenia in hypertensive patients. The higher the level of DII, the higher the risk of sarcopenia in hypertensive patients.

## Introduction

Hypertension is a disease characterized by elevated blood pressure that can cause damage to multiple target organs (1) and is currently thought to be an inflammation-related disease (2). Many studies have found that tumor necrosis factor alpha (TNF-α), C-reactive protein (CRP), chemokine and other inflammatory markers increase abnormally in hypertensive patients (3–5). Interestingly, activation of these inflammatory markers may interfere with cellular protein synthesis via the nuclear factor kappa-B (NF-κB) pathway, contributing to the development of sarcopenia (6). The activation of NOD-like receptor protein 3 (NLRP3) induced by inflammation is also one of the important pathways leading to the decline of muscle fibers (7). Sarcopenia is a degenerative disorder that is estimated to affect 50 million individuals globally is becoming increasingly prevalent (8). Research has demonstrated that people with hypertension are more likely to suffer from sarcopenia (9, 10), and those with both conditions have been found to be at greater risk of cognitive impairment (11), falling incidents (10), and albuminuria (12). Hence, it is necessary to prevent sarcopenia in hypertensive patients.

Given the association between inflammation and hypertension and sarcopenia, controlling inflammation may be a potential intervention to prevent sarcopenia in hypertensive patients. Diet is one of the important measures to control inflammation throughout the body. Energy, saturated fats and trans fats in foods increase levels of markers of inflammation throughout the body (TNF-α, CRP and IL-6) (13, 14). Vitamin E and omega-3 fatty acid intake were associated with decreased levels of markers of inflammation throughout the body (15). However, previous studies have proposed that individual dietary components are difficult to assess overall levels of dietary inflammation in patients due to the diversity of foods (16). In order to assess the overall level of dietary inflammation in patients, previous studies constructed dietary inflammatory index (DII) where high levels of DII represent higher inflammatory dietary potential, low levels of DII represent higher anti-inflammatory dietary inflammatory potential (17). Due to chronic kidney disease (CKD) and Crohn’s disease are both associated with inflammation, previous studies have used DII to assess the risk of developing sarcopenia in these patients (18, 19). However, few studies have examined the relationship between DII and sarcopenia in hypertensive patients.

The purpose of our study was to examine whether the risk of sarcopenia differs among hypertensive patients with different DII levels, and to provides some insights into the prevention of sarcopenia in hypertensive patients.

## Methods

### Study population

**Figure 1:**
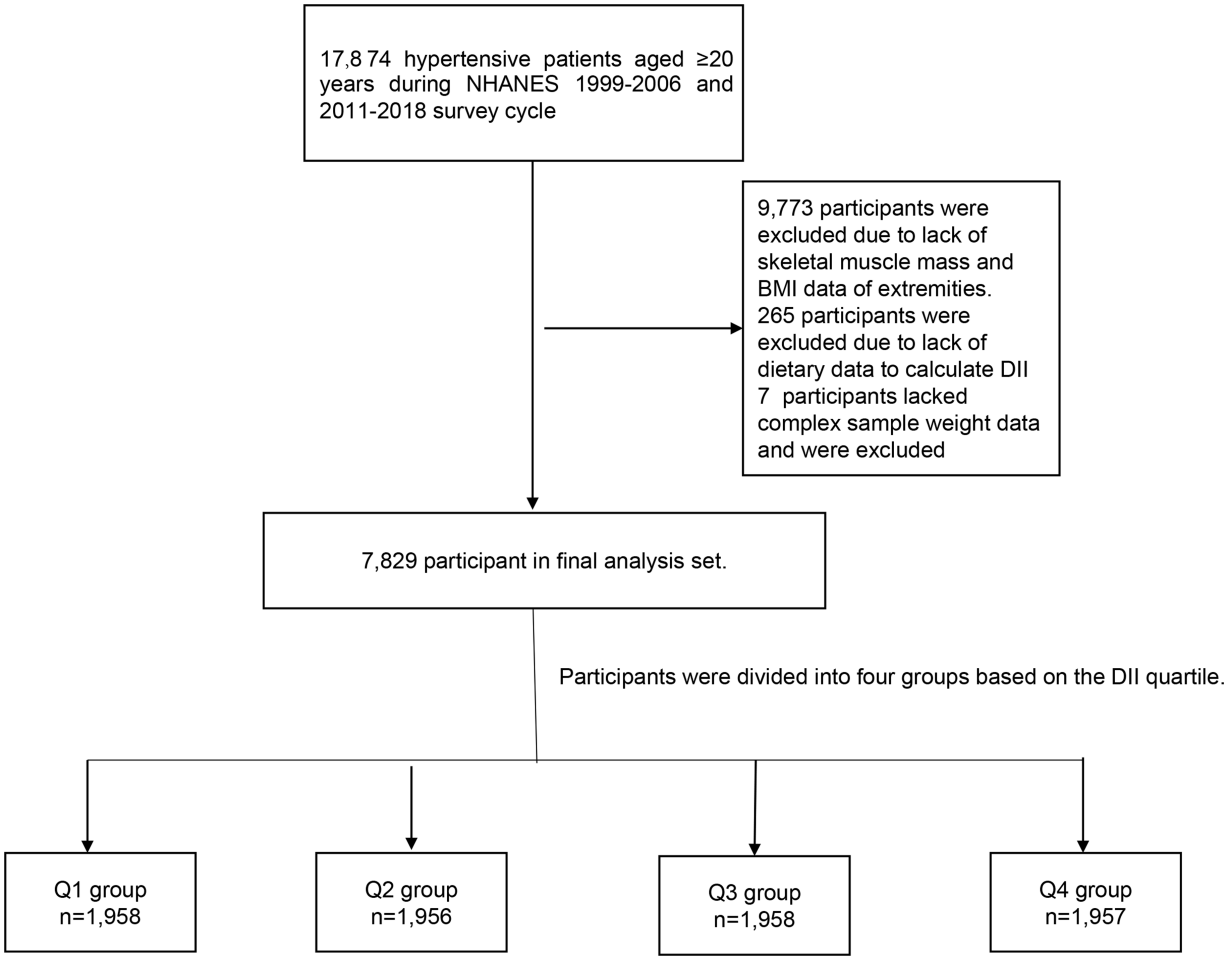
Flowchart of the study design.

The National Health and Nutrition Examination Survey (NHANES) is a comprehensive research project intended to evaluate the health and nutrition status of adults and children in the United States. Sampling approximately 5,000 individuals from various counties across the country every 2 years. Each participant was assigned a different sampling weight. After a complex sampling weighted analysis, these participants were able to represent the entire U.S. population. Due to the lack of skeletal muscle mass data between 2007 and 2010, the study was limited to participants from the years 1999 to 2006 and 2011 to 2018. In NHANES 1999–2006 and 2011–2018, there were 17,874 hypertensive patients over 20 years of age. A total of 9,773 participants lacking skeletal muscle mass and body mass index (BMI) data were deleted. 265 participants lacked dietary data to calculate DII and were excluded. 7 participants lacked dietary weight data and were excluded. After excluding these people, 7,829 people were eventually included in our study (Figure 1).

### Definition of hypertension

Hypertension is diagnosed according to the following three items: 1. According to the query posed in the NHANES: “Has a doctor ever told you that you have hypertension?,” and “Whether you are taking blood pressure medication,” those who answered “yes” were deemed to be hypertensive. 2. the systolic blood pressure (SBP) was measured in the mobile examination center and during home examinations on all eligible individuals using a mercury sphygmomanometer, participants with SBP greater than 140 mmHg or diastolic blood pressure (DBP) higher than 90 mmHg were regarded as hypertensive. In the event that the patient has multiple blood pressure readings, the average is used to make a diagnosis of hypertension. 3. Based on patient self-reported prescriptions, patients are considered hypertensive if they are currently taking calcium channel blockers (CCBS), beta blockers, diuretics, angiotensin-converting enzyme inhibitors, and/or angiotensin II receptor blockers (ACEIs/ ARBs). Participants who met one of these criteria were considered to have high blood pressure. This is consistent with previous research (20).

### Primary outcome

The primary endpoint was sarcopenia. Dual-energy X-ray absorptiometry (DXA) whole-body scans were used to appendicular skeletal muscle mass was measured using DXA. Whole body DXA scans were taken with a Hologic QDR-4500A fanbeam densitometer (Hologic, Inc., Bedford, Massachusetts). Hologic software version 8.26:a3* was used to administer all scans. Further details of the DXA examination protocol are documented in the Body Composition Procedures Manual located on the NHANES website: (https://search.cdc.gov/search/index.html?query=DXA&siteLimit=NCHS&dpage=1).

As recommended by the Foundation for National Institutes of Health Osteoarthritis Biomarkers study (FNIH), use the ratio of total appendicular skeletal muscle mass (in kg) to BMI (kg/m^2^) to determine if a patient has sarcopenia. The cut-off values for the diagnosis of sarcopenia were not identical (0.789 for men and 0.512 for women) due to physiological differences between men and women (21). This cut-off value was obtained by classification and regression tree (CART) analysis in previous studies (22). Many studies have used this standard to define sarcopenia (23–25).

### Calculation of the DII

Dietary inflammation index was designed as an exposure variable. The dietary data in NHANES were obtained by a 24 h dietary recall interview at the mobile examination center (MEC). In our study, carbohydrates, protein, total fat, alcohol, fiber, cholesterol, saturated fatty acids (SFAs), monounsaturated fatty acids (MUFAs), polyunsaturated fatty acids (PUFAs), omega-3 fatty acids, omega-6 fatty acids, niacin, vitamin A, vitamin B1, vitamin B2, vitamin B6, vitamin B12, vitamin C, vitamin D, vitamin E, iron, magnesium, zinc, selenium, folic acid, beta-carotene, caffeine, and energy were used to calculate DII. DII for each nutrient or dietary ingredient = [(daily intake of that nutrient or dietary ingredient -global *per capita* daily intake of that nutrient or dietary ingredient)/that nutrient or dietary ingredient Standard deviation of global *per capita* daily intake] x inflammatory effect index of that nutrient or dietary ingredient, and the sum of DII of each nutrient or dietary ingredient was the total DII score of individual study subjects (26). The anti-inflammatory or proinflammatory parameters of each food can be looked up in the study of Nitin Shivappa et al. (17).

### Confounding variable

The selection of confounding variables was determined based on previous studies. Studies have shown that these variables affect the occurrence and development of sarcopenia and need to be adjusted by incorporating regression models (27–30).

Age, sex, race, education level, poverty income ratio (PIR), smoking status and alcohol use were self-reported by participants. BMI was calculated based on the height and weight of the participants. Diagnosis of comorbidities was based on an affirmative response to the question “Has a doctor or other health professional ever told you that you had diabetes mellitus (DM), CKD, cardiovascular disease [CVD (include coronary heart disease, congestive heart failure, heart attack, stroke and angina)]?.” Participants were also considered diabetic if they were being treated for diabetes, or had a hemoglobin a1c (HbA1c) of 6.5 percent or more. In addition, participants with estimated glomerular filtration rate (eGFR) <60 mL/min/1.73 m^2^ and/or randomized urinary albumin/creatinine ratio (ACR) >30 mg/g were also considered patients with CKD (31). Laboratory measurements, such as triglycerides (TC), total cholesterol (TG) and C-reactive protein (CRP) were collected using automated hematological analysis equipment. Urine albumin was measured by fluorescence immunoassay. Urinary creatinine was measured using Roche/Hitachi modular P chemical analyzer Detailed procedures for obtaining laboratory measurements were provided in a document on the website^1^ of the National Center for Health Statistics. In addition, muscle loss caused by statins, sulfonylureas and glycinates was also defined as confounding variables (32–34). Self-reported prescription data was used to determine if the patient was taking these medications, which were defined as other drugs.

### Method of grouping

The independent variable DII was included as a grouping variable for the purpose of the study, which is consistent with previous studies (35–37). Patients were divided into four groups based on the quartile of DII: Group 1 (DII < 0.35), Group 2 (0.35 ≤ DII <1.82), Group 3 (1.82 ≤ DII <2.90), Group 4 (DII ≥ 2.90).

### Statistical analyses

According to the National Health and Nutrition Examination Survey (NHANES) recommended weights, the weights for specific groups were calculated. Continuous variables were expressed as the mean (standard error), and categorical variables were presented as counts (percentages). Baseline characteristics between the different groups were compared using an analysis of variance (ANOVA) for continuous variables, and a χ2 test for categorical variables.

We conducted logistic regression analyses to assess the association between DII and sarcopenia. All statistical analyses were performed with complex sampling weighted analysis using the weights recommended by NHANES. In order to enhance the robustness of the results, three models were analyzed. Model 1 was the unadjusted model. Model 2 was adjusted for age, gender, and race. Model 3 was fully adjusted for potential confounders, including age, gender, race, smoking status, drinking status, education level, PIR, BMI, TG, TC, the use of antihypertensive drugs, other drugs, DM, CVD, and CKD. To investigate the potential non-linear relationship between DII and sarcopenia, a regression cubic spline (RCS) analysis was also conducted. The adjustment variables for the RCS are consistent with Model 3. In addition, we stratified the analysis by age, sex, and antihypertensive drug use, and analyzed whether there was an interaction between DII and these subgroups.

All data analyses were performed by using the Survey package in R software (version 4.2.2; R Foundation for Statistical Computing, Vienna, Austria). A two-sided *p*-value <0.05 indicated significance for all analyses.

## Results

### Participant characteristics

The baseline clinical characteristics are reported in Table 1. In this study, 7,829 patients with hypertension were enrolled, with an average age of 51.4 (0.3) years, and 3,872 (47.9%) of the participants being female. In total, 1,871 (18.7%) had CVD, 1,737 (17.4%) had DM, 4,111 (49.2%) were routinely taking antihypertensive drugs, and 1,352 (17.3%) had sarcopenia. Participants were divided into four groups based on the DII quartile [Q1 (*n* = 1,958); Q2 (*n* = 1,956); Q3 (*n* = 1,958); Q4 (*n* = 1,957)]. There was a statistically significant difference in mean age [Q1: 50.9(0.5) vs. Q2: 51.6(0.5) vs. Q3: 52.4(0.4) vs. Q4: 50.9(0.4), *p* = 0.017] between the four groups. Furthermore, those with higher DII were more likely to be female [Q1: 33.6% vs. Q2: 46.6% vs. Q3: 53.1% vs. Q4: 61.1%, *p* < 0.001] and had lower education level [education level > 12; Q1: 64.5% vs. Q2: 58.1% vs. Q3: 48.5% vs. Q4: 45.8%, *p* < 0.001] and PIR [Q1: 3.4 (0.1) vs. Q2: 3.1 (0.1) vs. Q3: 2.9 (0.1) vs. Q4: 2.6 (0.1), *p* < 0.001] and were less likely to drink [Q1: 75.2% vs. Q2: 69.2% vs. Q3: 67.3% vs. Q4: 61.5%, *p* < 0.001]. Additionally, those with higher DII had higher CRP [Q1: 0.37(0.02) vs. Q2: 0.48(0.04) vs. Q3: 0.47(0.03) vs. Q4: 0.60(0.03), *p* < 0.001] and a higher prevalence of DM [Q1: 15.1% vs. Q2: 16.4% vs. Q3: 17.9% vs. Q4: 20.8%, *p* = 0.005], CKD [Q1: 15.4% vs. Q2: 18.8% vs. Q3: 19.2% vs. Q4: 20.0%, *p* = 0.003], and CVD [Q1: 10.9% vs. Q2: 12.7% vs. Q3: 13.4% vs. Q4: 15.1%, *p* = 0.009]. No significant differences were found in smoking (*p* = 0.609), SBP (*p* = 0.249), TG (*p* = 0.502), TC (*p* = 0.313), use of antihypertensive drugs (*p* = 0.198) and other drugs (*p* = 0.575) among the four groups. Further detailed information is shown in Table 1.

**Table 1:** Baseline study population characteristics (weighted).

Characteristics	Overall (*N* = 7,829)	Q1 group (*N* = 1,958)	Q2 group (*N* = 1,956)	Q3 group (*N* = 1,958)	Q4 group (*N* = 1,957)	*p*-value
Age, years	51.4(0.3)	50.9(0.5)	51.6(0.5)	52.4(0.4)	50.9(0.4)	0.017
Female, *n* (%)	3,872(47.9)	699(33.6)	917(46.6)	1,036(53.1)	1,220(61.1)	< 0.001
Race, *n* (%)						< 0.001
Mexican American	1,329(6.6)	355(7.3)	349(7.3)	315(5.3)	310(6.3)	
Non-Hispanic Black	1,961(13.6)	389(10.1)	433(12.3)	544(15.1)	595(17.5)	
Non-Hispanic White	3,478(68.2)	905(70.3)	899(68.7)	869(68.9)	805(64.3)	
Other Hispanic	463(5.1)	124(5.1)	115(5.0)	106(4.8)	118(5.6)	
Other Race	598(6.5)	185(7.2)	160(6.7)	124(5.9)	129(6.2)	
Education, *n* (%)						< 0.001
<12	2,323(19.5)	469(14.3)	549(18.0)	635(22.5)	670(24.0)	
12	1,886(25.9)	421(21.2)	449(24.0)	500(29.0)	516(30.2)	
>12	3,612(54.7)	1,067(64.5)	955(58.1)	820(48.5)	770(45.8)	
DII	1.42(0.04)	−0.92(0.04)	1.11(0.01)	2.37(0.01)	3.57(0.02)	< 0.001
SBP, mmHg	134.6(0.4)	133.8(0.8)	135.1(0.6)	135.6(0.6)	134.2(0.6)	0.249
Smoking, *n* (%)	3,916(51.4)	977(50.7)	955(50.1)	993(52.0)	991(53.1)	0.609
Drinking, *n* (%)	4,879(68.6)	1,339(75.2)	1,273(69.2)	1,194(67.3)	1,073(61.5)	< 0.001
BMI, kg/m^2^	30.00(0.12)	29.62(0.19)	29.53(0.20)	30.60(0.21)	30.32(0.23)	< 0.001
TG, mmol/L	1.98(0.03)	2.05(0.08)	1.94(0.05)	1.97(0.05)	1.92(0.04)	0.502
TC, mmol/L	5.31(0.02)	5.27(0.03)	5.29(0.04)	5.34(0.04)	5.35(0.04)	0.313
CRP, mg/dL	0.48(0.02)	0.37(0.02)	0.48(0.04)	0.47(0.03)	0.60(0.03)	< 0.001
PIR	3.00(0.04)	3.35(0.06)	3.10(0.06)	2.86(0.06)	2.61(0.06)	< 0.001
DM, *n* (%)	1,737(17.4)	402(15.1)	426(16.4)	453(17.9)	456(20.8)	0.005
CKD, *n* (%)	1,871(18.7)	393(15.4)	481(18.8)	484(19.2)	513(22.0)	0.003
CVD, *n* (%)	1,218(12.9)	269(10.9)	287(12.7)	312(13.4)	350(15.1)	0.009
Antihypertensive drug, *n* (%)	4,111(49.2)	1,008(46.5)	1,012(49.4)	1,040(50.7)	1,051(50.8)	0.198
Other drugs, *n* (%)	1,643(20.3)	404(19.1)	429(20.6)	410(21.6)	400(19.9)	0.575
Sarcopenia, *n* (%)	1,352(17.3)	265(9.4)	309(10.9)	349(15.3)	429(19.3)	< 0.001

Values are, *n* (%) or mean (SE).

DII, Dietary inflammatory index; SBP, systolic blood pressure; BMI, body mass index; TG, triglyceride; TC, total cholesterol; PIR，poverty income ratio; DM, diabetes mellitus; CKD, Chronic kidney disease. CVD, cardiovascular disease.

### The association between DII and sarcopenia

**Figure 2:**
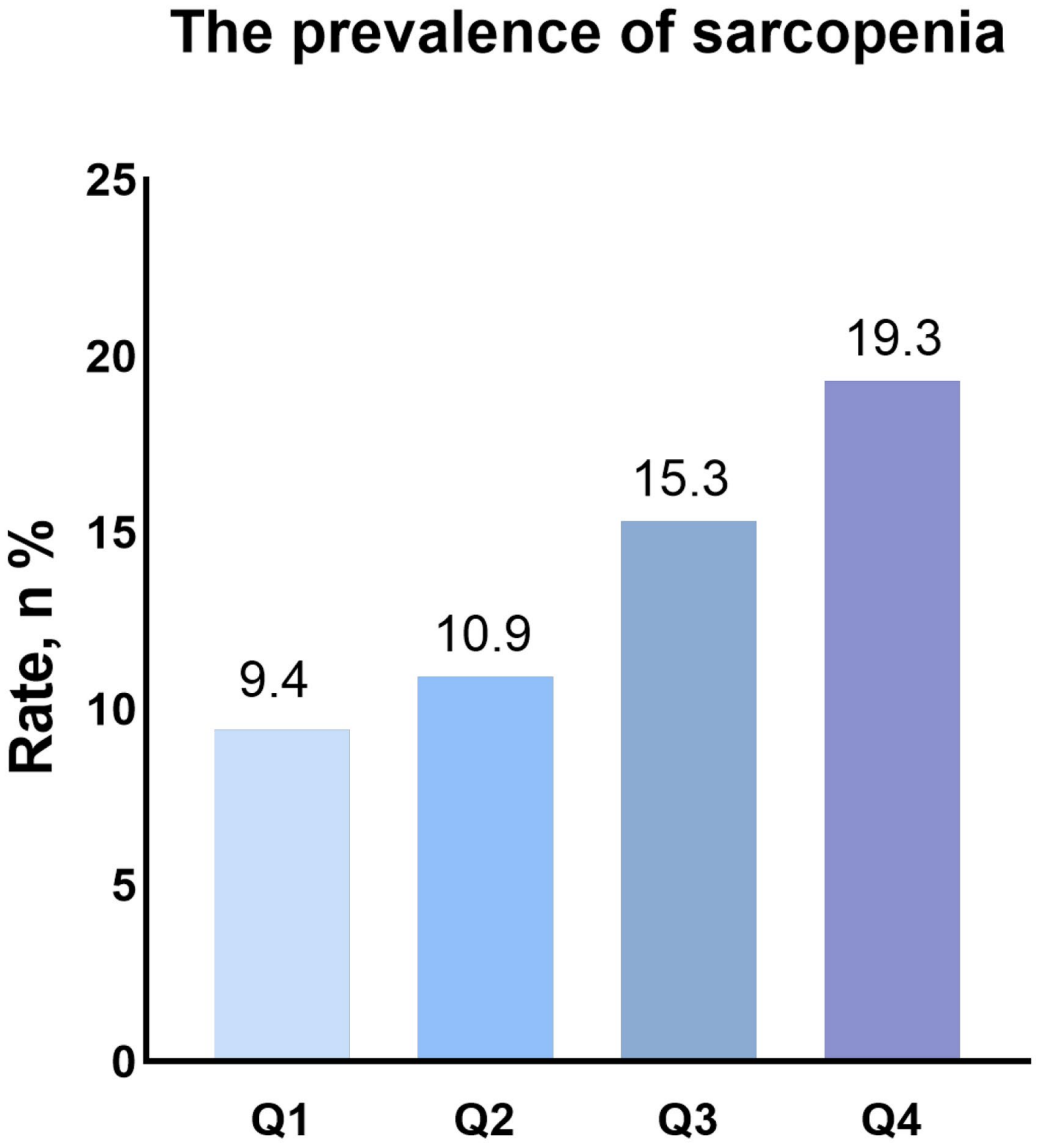
Prevalence of sarcopenia in different groups (weighted).

As shown in Figure 2, the group with higher DII levels had a higher prevalence of sarcopenia (Q1 group:9.4% vs. Q2 group:10.9% vs. Q3 Group: 15.3% vs. Q4 group: 19.3). Univariate logistic regression analysis showed that DII (OR: 1.22, 95% CI: 1.15–1.30, *p* < 0.001) was significantly associated with sarcopenia in patients with hypertension. Compared with Q1 group, the group with higher DII levels had a higher risk of having sarcopenia (Q2: OR: 1.17, 95%CI: 0.97–1.51, *p* = 0.214; Q3: OR: 1.74, 95%CI: 1.35–2.24, *p* < 0.001; Q4: OR: 2.29, 95%CI: 1.75–3.01, *p* < 0.001). After adjusting for age, sex, race, smoking status, drinking status, education, PIR, BMI, TG, TC, antihypertensive drug, other drugs, DM, CVD and CKD, the association between DII (OR: 1.22, 95% CI: 1.13–1.32, *p* < 0.001) and sarcopenia did not change. Patients with higher DII have a higher risk of having sarcopenia (Q2: OR: 1.23, 95%CI: 0.89–1.72, *p* = 0.209; Q3: OR: 1.68, 95%CI: 1.20–2.35, *p* = 0.003; Q4: OR: 2.43, 95%CI: 1.74–3.39, *p* < 0.001) (Table 2).

**Table 2:** The association between DII and sarcopenia in patients with hypertension (weighted).

Variable		Model 1		Model 2		Model 3	
		OR (95%CI)	*p*-value	OR (95%CI)	*p*-value	OR (95%CI)	*p*-value
Continuous variables							
DII		1.22(1.15–1.30)	<0.001	1.29(1.21–1.37)	<0.001	1.22(1.13,1.32)	<0.001
Categorical variable	Event/All population						
Q1 group	265/1958	Ref		Ref		Ref	
Q 2 group	309/1956	1.17(0.91–1.51)	0.214	1.21(0.93–1.58)	0.161	1.23(0.89–1.72)	0.209
Q 3 group	349/1958	1.74(1.35–2.24)	<0.001	1.95(1.49–2.55)	<0.001	1.68(1.20–2.35)	0.003
Q 4 group	429/1957	2.29(1.75–3.01)	<0.001	2.88(2.15–3.85)	<0.001	2.43(1.74–3.39)	<0.001

Model 1: Not adjusted.

Model 2: Adjusted by age, gender, race/ethnicity.

Model 3: Adjusted by age, gender, race/ethnicity, smoking status, drinking status, education, PIR, BMI, TG, TC, antihypertensive drug, other drugs, DM, CVD, CKD.

### Subgroup analysis

**Figure 3:**
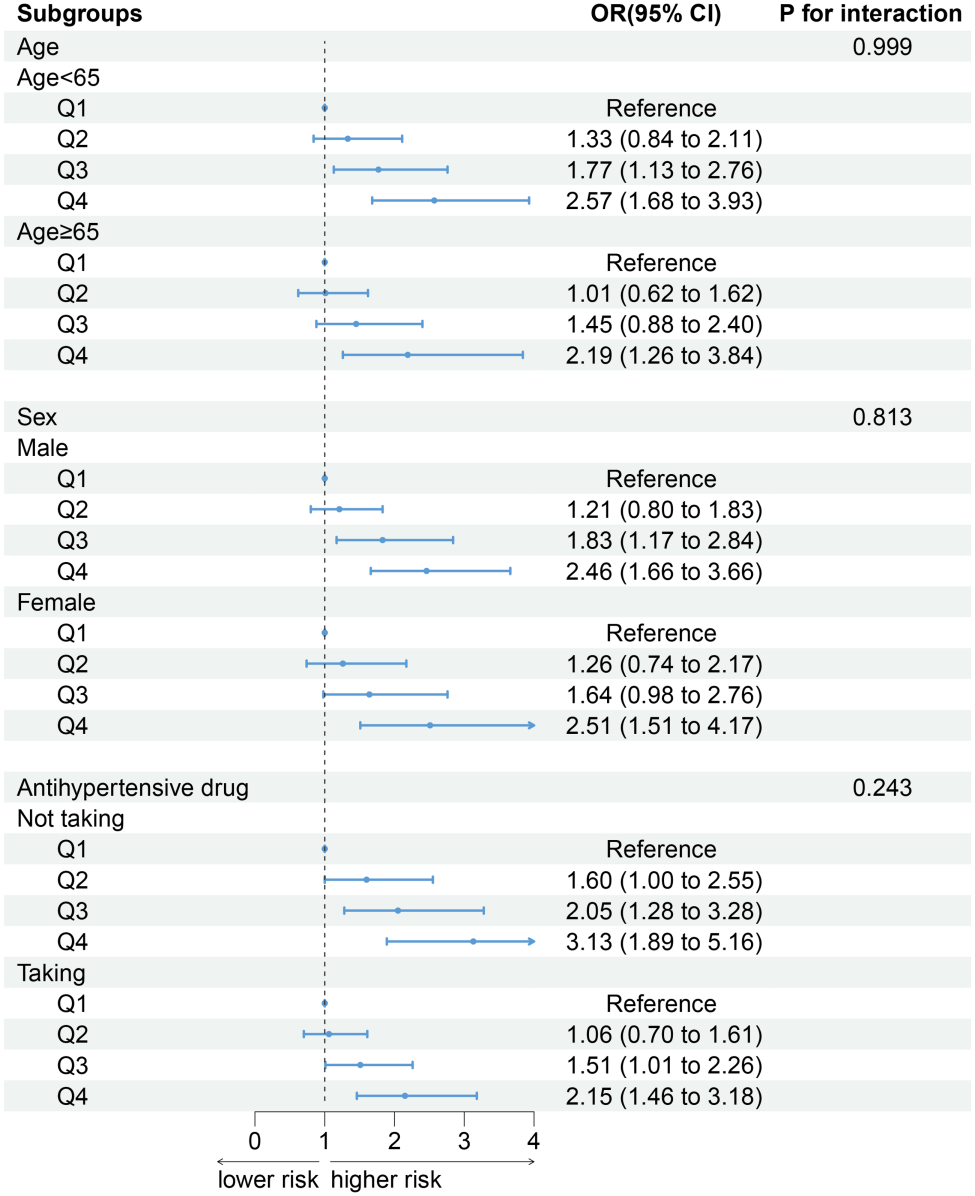
Association between DII and sarcopenia by selected subgroups (weighted).

After stratifying the participants according to age (*p* for interaction = 0.999), gender (*p* for interaction = 0.813) and antihypertensive drug (*p* for interaction = 0.243), the association between DII and sarcopenia did not change. Compared with Q1 group, the groups with higher DII have higher the risk of developing sarcopenia (Figure 3).

### Regression cubic splines

**Figure 4:**
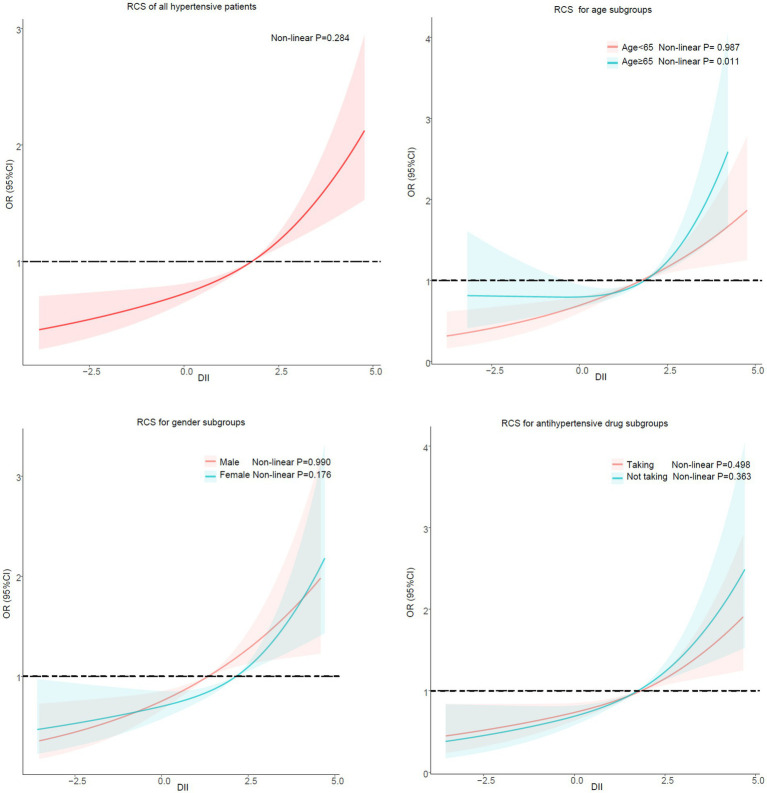
Potential nonlinear relationship between DII and sarcopenia (weighted).

After stratifying the participants according to gender and antihypertensive drug, no potential non-linear relationship was observed between DII and sarcopenia in hypertensive patients. However, there was a non-linear relationship between DII and sarcopenia in hypertensive patients in the subgroup ≥65 years of age (Non-linear *p* = 0.011) but not in the subgroup <65 years of age (Non-linear *p* = 0.987) (Figure 4).

## Discussion

In this cross-sectional study, our results show that DII is associated with the risk of sarcopenia in hypertensive patients. The higher the DII score, the higher the patient’s risk of sarcopenia. There was no significant change in the association between DII and sarcopenia in hypertensive patients after stratified analysis based on age, sex, and antihypertensive drugs.

Inflammation is one of the important ways of the occurrence and development of sarcopenia (38). Chronic inflammation can accelerate protein breakdown and promotes sarcopenia by activating the ubiquitin-proteasome system, caspase 3, lysosome, and myostatin (39). In addition, as stated in the preface, hypertension is currently recognized as an inflammation-related disease, and multiple inflammatory markers have been shown to be abnormally elevated in hypertensive patients (3–5), Activation of these inflammatory markers promotes sarcopenia through NF-κB and NLRP3 (6, 7). This provides a theoretical basis for preventing sarcopenia by regulating the inflammation level in hypertensive patients.

Diet is an effective measure to improve systemic inflammation. Studies have constructed DII based on the anti-inflammatory and pro-inflammatory levels of foods to assess dietary inflammatory potential (17). Evidence has demonstrated that the DII is significantly associated with various markers of systemic inflammation, such as C-reactive protein (CRP), neutrophil-to-lymphocyte ratio (NLR), tumor necrosis factor-alpha (TNF-α), and procalcitonin (40, 41). Nilufal Shoei et al. found that high DII is associated with an increased risk of hypertension (42). In addition, Cao et al. found that DII was associated with the risk of all-cause mortality in hypertensive patients, and the higher the DII, the higher the all-cause mortality in hypertensive patients (43). Our study links DII to sarcopenia in hypertensive patients. The results showed that the higher the DII, the higher the risk of sarcopenia in hypertensive patients. Previous studies have shown that DII was associated with sarcopenia in the elderly, CKD patients, and Crohn’s patients. The higher the DII, the higher the risk of sarcopenia (18, 19, 44). The results of these studies are consistent with ours.

Our results demonstrate that DII is significantly correlated with the risk of sarcopenia in hypertensive patients. Those with higher DII scores are more likely to suffer from sarcopenia. DII is an index to evaluate the dietary inflammatory potential of patients. Pro-inflammatory diet may promote the occurrence and development of myopathy by aggravating systemic inflammation levels in hypertensive patients to activate a variety of enzyme systems to accelerate muscle breakdown (39, 45). However, due to the cross-sectional nature of this study, we can only make this assumption, and further prospective investigations are needed to verify our hypothesis.

In subgroup analyses stratified by age, gender and antihypertensive drug, the result of regression analysis was in line with the primary findings. High levels of DII was an independent risk factor for sarcopenia in hypertensive patients. Our results are consistent with previous studies (46, 47). We further examined the potential nonlinear correlation between DII and sarcopenia by using restricted regression cubic splines. No potential non-linear relationship was observed in the RCS stratified by gender and antihypertensive medications. However, a non-linear connection between DII and sarcopenia in individuals aged 65 or above was observed in our findings. Too low DII had no significant preventive effect on patients with sarcopenia. It’s not impossible to explain. DII was calculated and briefly explained as follows: DII for each nutrient or dietary ingredient = [(daily intake of that nutrient or dietary ingredient -global *per capita* daily intake of that nutrient or dietary ingredient)/that nutrient or dietary ingredient Standard deviation of global *per capita* daily intake] x inflammatory effect index of that nutrient or dietary ingredient, and the sum of DII of each nutrient or dietary ingredient was the total DII score of individual study subjects (26). Patients with low DII scores also have low intakes of various dietary substances (including energy, protein, fat, etc), which can lead to malnutrition (48, 49), a high risk factor for sarcopenia (50–52). Older people are known to be at high risk for malnutrition (53), so we speculate that in elderly patients with low DII, the risks of malnutrition may mask the benefits of an anti-inflammatory diet. But further prospective studies are needed to confirm our suspicions.

In this cross-sectional study, our results suggest that pro-inflammatory diet is an independent risk factor for sarcopenia in hypertensive patients. But consider that low DII scores are associated with the intake of various nutrients, which can lead to malnutrition and an increased risk of sarcopenia. Therefore, people at nutritional risk, such as the elderly, should pay attention to the intake of energy, protein, fat and other substances while maintaining an anti-inflammatory diet to prevent sarcopenia.

## Limitations

There were some study limitations. First, it was subject to the limitations inherent of retrospective analysis. The relationship between DII and sarcopenia could only be interpreted as a correlation, rather than as a causal relationship. Second, Previous studies calculated DII based on 45 foods. Since only 28 dietary data from NHANES could be used to calculate DII, our study calculated the sum of DII for only 28 foods. However, previous studies have confirmed that DII calculated using only 28 foods does not affect the predictive effectiveness of DII (54). Third, Due to the limited data on grip strength in NHANES, the diagnosis of sarcopenia in our study relied solely on muscle mass without combining grip strength. Further prospective studies are needed to confirm our results.

## Conclusion

DII is associated with the risk of sarcopenia in hypertensive patients. The higher the DII score, the higher the risk of sarcopenia. Low DII may not have a positive effect on the prevention of sarcopenia in hypertensive individuals older than 65 years.

## Data availiability statement

The raw data supporting the conclusions of this article will be made available by the authors, without undue reservation.

## Ethics statement

Ethical review and approval was not required for the study on human participants in accordance with the local legislation and institutional requirements. Written informed consent for participation was not required for this study in accordance with the national legislation and the institutional requirements.

## Author contributions

LC designed the research and is the guarantor of this work and, as such, had full access to all the data in the study and takes responsibility for the integrity of the data and the accuracy of the data analysis. JT conducted the analysis and wrote the first draft of the paper. SS, YuL, JX, YZ, BW, YiL, KC, GL, and LC revised the manuscript. All authors contributed to the article and approved the submitted version.

## Funding

This research was funded and supported by Longyan City Science and Technology Plan Project (grant numbers: 2021LYF17309 and 2022LYF17026).

## Conflict of interest

The authors declare that the research was conducted in the absence of any commercial or financial relationships that could be construed as a potential conflict of interest.

## Publisher’s note

All claims expressed in this article are solely those of the authors and do not necessarily represent those of their affiliated organizations, or those of the publisher, the editors and the reviewers. Any product that may be evaluated in this article, or claim that may be made by its manufacturer, is not guaranteed or endorsed by the publisher.

## Footnotes

^1^https://wwwn.cdc.gov/nchs/data/nhanes/2009-2010/manuals/lab.pdf
